# Preparedness and Response Activities of the VA Home-Based Primary Care Program Around the Fall 2017 Hurricane Season

**DOI:** 10.1093/geroni/igaa057.1982

**Published:** 2020-12-16

**Authors:** Tamar Wyte-Lake, Claudia Der-Martirosian, Aram Dobalian

**Affiliations:** 1 US Department of Veterans Affairs, North Hills, California, United States; 2 Veterans Emergency Management Evaluation Center, North Hills, California, United States

## Abstract

Individuals aged seventy-five or older, who often present with multiple comorbidities and decreased functional status, typically prefer to age in their homes. Additionally, as in-home medical equipment evolves, more medically vulnerable individuals can receive care at home. Concomitantly, large-scale natural disasters disproportionally affect both the medically complex and the older old, two patient groups responsible for most medical surge after a disaster. To understand how to ameliorate this surge, we examined the activities of the nine US Department of Veterans Affairs Home-Based Primary Care programs during the 2017 Atlantic Hurricane Season. These and similar programs under Medicare connect the homebound to the healthcare community. Study findings support early implementation of preparedness procedures and intense post-Hurricane patient tracking as a means of limiting reductions in care and preventing significant disruptions to patient health. Engaging with home-based primary care programs during disasters is central to bolstering community resilience for these at-risk populations.

